# Single-cell sequencing and spatial transcriptomics reveal the evolution of glucose metabolism in hepatocellular carcinoma and identify G6PD as a potential therapeutic target

**DOI:** 10.3389/fonc.2025.1553722

**Published:** 2025-03-25

**Authors:** Deyang Xi, Yinshuang Yang, Jiayi Guo, Mengjiao Wang, Xuebing Yan, Chunyang Li

**Affiliations:** ^1^ Graduate School, Xuzhou Medical University, Xuzhou, Jiangsu, China; ^2^ Department of Infectious Diseases, The Affiliated Hospital of Xuzhou Medical University, Xuzhou, Jiangsu, China

**Keywords:** hepatocellular carcinoma, metabolic reprogramming, carbohydrate metabolism, prognostic biomarker, bioinformatics

## Abstract

**Background:**

Glucose metabolism reprogramming provides significant insights into the development and progression of malignant tumors. This study aims to explore the temporal-spatial evolution of the glucose metabolism in HCC using single-cell sequencing and spatial transcriptomics (ST), and validates G6PD as a potential therapeutic target for HCC.

**Methods:**

We collected single-cell sequencing data from 7 HCC and adjacent non-cancerous tissues from the GSE149614 database, and ST data from 4 HCC tissues from the HRA000437 database. Pseudotime analysis was performed on the single-cell data, while ST data was used to analyze spatial metabolic activity. High-throughput sequencing and experiments, including wound healing, CCK-8, and transwell assays, were conducted to validate the role and regulatory mechanisms of G6PD in HCC.

**Results:**

Our study identified a progressive upregulation of PPP-related genes during tumorigenesis. ST analysis revealed elevated PPP metabolic scores in the central and intermediate tumor regions compared to the peripheral zones. High-throughput sequencing and experimental validation further suggested that G6PD-mediated regulation of HCC cell proliferation, migration, and invasion is likely associated with glutathione metabolism and ROS production. Finally, Cox regression analysis cofirmed G6PD as an independent prognostic factor for overall survival in HCC patients.

**Conclusion:**

Our study provides novel insights into the changes in glucose metabolism in HCC from both temporal and spatial perspectives. We experimentally demonstrated that G6PD regulates proliferation, migration, and invasion in HCC and propose G6PD as a prognostic marker and therapeutic metabolic target for the HCC.

## Introduction

Hepatocellular carcinoma (HCC) is the most prevalent malignant liver tumor, ranking as the sixth most common cancer and the third leading cause of cancer-related mortality worldwide (1, 2). Over the past few decades, the incidence of HCC has risen, particularly in the Asia-Pacific region and parts of Africa, where there is a high prevalence of hepatitis B and C viruses (3). With improved living standards, liver cancers arising from metabolic liver diseases are also on the rise (4). HCC is highly aggressive, and most patients are diagnosed at an advanced stage with a poor prognosis (5). Although targeted therapies have extended survival times to some extent, their high cost and severe side effects limit their widespread clinical use (6).

Cancer cells adapt their energy metabolism to optimize the rapid consumption and utilization of glucose to support their rapid proliferation and growth needs (7–9). This metabolic mode, known as the Warburg effect, entails a preference for glycolysis over oxidative phosphorylation even in the presence of adequate oxygen, thereby efficiently generating energy to promote tumor growth and survival (10, 11). Through metabolic reprogramming, cancer cells can rapidly synthesize intermediates such as nucleic acids, lipids, and proteins to continually support their proliferation and spread (12). Hence, targeting glucose metabolism—including glycolysis, the pentose phosphate pathway (PPP), and the TCA cycle—is considered an attractive approach to cancer therapy (13).

Reprogramming of glucose metabolism provides a better understanding of the onset and progression of malignancies, further clarifying the complexities of cancer (14, 15). This study aims to explore the evolution of glucose metabolism in HCC through detailed analysis using single-cell RNA sequencing (scRNA-seq) and spatial transcriptomics (ST) and to ascertain the role of G6PD in the malignant transformation of HCC, thereby providing suitable metabolic targets for drug development.

## Materials and methods

### Single-cell sequencing data acquisition and processing

We retrieved data from the GEO database, specifically dataset GSE149614, which included cancer and adjacent non-tumor tissues from 10 HCC patients. Patients 1 and 2, who only had cancer tissue samples without corresponding adjacent non-tumor tissues, were excluded from the analysis. We utilized the R packages “Seurat” and “SingleR” to analyze the scRNA-seq data (16, 17). Mitochondrial gene expression levels are typically associated with cellular health. When the proportion of mitochondrial genes exceeds 10%, it often indicates that the cell is under stress or undergoing apoptosis (18–20). To ensure the inclusion of high-quality cellular data, cells with gene counts outside the 2% to 98% percentile range and those with mitochondrial gene content exceeding 10% were excluded. Anomalies in patient 7’s cancer tissue, which only contained 489 cells, suggested clinical sample issues, leading to their exclusion. Ultimately, data from 7 cancer tissues and their corresponding adjacent non-tumor tissues were included. We normalized the scRNA-seq data using the “NormalizeData” function in the Seurat R package. The normalized data were then converted into Seurat objects, and the top 5000 variable genes were identified using the “FindVariableFeatures” function. Dimensionality reduction of the scRNA-seq data was performed using the “RunPCA” function for principal component analysis (PCA). To mitigate batch effects between samples, we applied the “harmony” R package. Cell clustering was accomplished using the “FindNeighbors” and “FindClusters” functions with a resolution parameter of 0.5, followed by visualization of the results using the t-SNE method. Cell type annotation was refined using the “SingleR” R package, which predicts cell types based on their correlation with a reference database, continuously eliminating the least correlated types (21).

### Pseudotime analysis

We employed the “Monocle2” R package to infer the developmental trajectory of our target cells through gene conversion into reverse graph embedding and dimensionality reduction techniques, arranging cells in a pseudotime sequence (22). To explore the evolutionary differentiation of glucose metabolism in HCC, we extracted a hepatocyte subgroup from all cells and conducted trajectory analysis using “Monocle2.” The “DDTree” method was used for dimensionality reduction of these cells. Cell ordering was performed using the “orderCells” function. The results were visually analyzed using the “plot_cell_trajectory” function to understand the dynamic changes in cellular states across the developmental continuum (23, 24).

### Acquisition and processing of spatial transcriptomics data

The ST data were sourced from the HRA000437 database (25). During the quality control phase, we eliminated genes expressed in fewer than 5 spots, spots with fewer than 300 detected features, and spots where mitochondrial gene content exceeded 10%. Normalization was performed using the SCTransform method with default parameters in the Seurat R package. Dimensionality reduction and clustering of the data were achieved using the RunPCA, FindNeighbors, FindClusters, and RunUMAP functions in Seurat.

Spatial data visualization was conducted using the “SpatialDimPlot” function, with the center of the tumor serving as the center for spatial plotting of transcriptomics cells. First, we calculated the distance of each cell from the tumor center using the formula (Distance = $\sqrt{{\left(x-x_{center}\right)}^{2}+{\left(y-y_{center}\right)}^{2}}$ ), Subsequently, we divided the spatial domain into three regions using tertiles to ensure that each region contained approximately one-third of the cells:

- First region (Central Core): Includes cells from the minimum distance up to the first tertile.
- Second region (Intermediate zones): Spans from the first to the second tertile.
- Third region (Out Periphery): Ranges from the second tertile to the maximum distance.

For quantifying metabolic activity at a single-cell resolution, we employed the “scMetabolism” R package, applying it to measure the metabolic activities across all hepatocytes (26).

### Cell transfection

HepG2 and Hep3B cells were cultured in Dulbecco’s Modified Eagle’s Medium (Gibco, USA) supplemented with 10% fetal bovine serum (Gibco, USA) and 1x penicillin/streptomycin (Biyuntian, China). All cultures were maintained at 37°C in a 5% CO_2_ incubator (Thermo Fisher Scientific, USA). Gene knockdown of G6PD was achieved using small interfering RNA (siRNA), specifically si-G6PD#1 and si-G6PD#2. The mRNA levels of G6PD were quantified relative to β-Actin mRNA levels using RT-qPCR and Western Blot (WB) to assess transfection efficiency. For the WB analysis, the membrane strips were initially trimmed and subsequently individually hybridized with antibodies, with four markers retained on each membrane. The full WB image is a composite created after antibody hybridization. Relative gene expression levels were calculated using the 2^-ΔΔCt method. All primers were supplied by Sangon Biotech (Sangon Biotech, China), with sequences listed in **Supplementary Table 1**. This study was reviewed and approved by the Ethics Committee of the Affiliated Hospital of Xuzhou Medical University (No: XYFY2024-KL283-01).

### CCK-8 Assay, wound healing assay, and transwell assay

**CCK-8 Assay:** 1×10^3 cells were cultured in each well of a 96-well plate. A 1% CCK-8 solution (Meilunbio, China) was added to each well, and the cells were incubated at 37°C in a 5% CO2 incubator for 1 hour to assess cell proliferation. Absorbance at OD450 was measured daily from day 1 to day 7 using a microplate reader (Synergy H1, USA).

Wound healing assay: Cells were cultured in 6-well plates until 95% confluence. A sterile 200 μl plastic pipette tip was used to scratch a straight line in each well. The wells were gently washed twice with PBS to remove unattached cells and debris. Cell migration was observed at 0h and 48h. Images of the scratch wounds were taken at 0 hours and 48 hours using Image J software, and the cell migration rate was calculated (Migration rate= (Width at 48h−Width at 0h)/Width at 48h).

Transwell assay: Treated cells (2×10^5) were seeded into the upper chamber of a 24-well plate and incubated for 48 hours. To assess cell migration and invasion capabilities, the upper surface of the insert was either left uncoated or pre-coated with matrix gel solution (LYNJUNE, China). After removing cells from the surface, the remaining cells on the bottom were fixed with 4% paraformaldehyde and stained with 0.1% crystal violet (VICMED, China).

### G6PD knockdown HepG2 Cell Line construction and RNA sequencing

Cells were seeded in 24-well plates for gene knockdown experiments. Using Lipofectamine 2000, recombinant lentivirus particles were transfected into 293T cells to produce lentivirus. The plasmids contained G6PD-specific shRNA (5’-GCCGTGTACACCAAGATGA-3’). HepG2 cells were transfected with lentiviral particles and selected with puromycin to generate stable cell lines. Total RNA was isolated and purified using TRIzol, and RNA libraries were sequenced on the Illumina NovaseqTM 6000 platform (LC Bio Technology CO., Ltd., Hangzhou, China) according to standard procedures. Differential analysis was performed using DESeq2, and GSEA enrichment analysis was conducted using the “clusterProfiler” R package (27).

### NADP/NADPH measurement and ROS detection

NADP/NADPH measurement: NADP/NADPH levels were measured using the BIOSS (AK302) kit. Cells were collected into centrifuge tubes and treated with alkaline/acidic extraction solution. After centrifugation, the supernatant was collected, and absorbance was measured at OD570 nm using a microplate reader (Synergy H1, USA).

ROS Detection: DCFH-DA (Beyotime, China) was diluted in serum-free medium at a 1:1000 ratio to achieve a final concentration of 10 µM. After removing the culture medium from the cells, they were washed with PBS, and then 1.5 mL of the diluted DCFH-DA was added to each well. The cells were incubated at 37°C in a 5% CO2 incubator for 20 minutes. After the incubation, cells were washed three times with serum-free medium, fixed with paraformaldehyde, and the fluorescence intensity was measured using a flow cytometer (FACScanto II*, USA) with an excitation at 488 nm and emission at 525 nm. Analysis was performed using FlowJo software.

### Pan-cancer analyses of differential G6PD expression and survival analysis

We collected G6PD mRNA levels and clinical information from tumor and normal tissues across 33 cancer types available in the TCGA database. Differential expression of the gene was analyzed using the ‘ggplot2’ R package. Bar charts were utilized to display the expression level differences across various cancers.

For survival analysis, univariate Cox regression was conducted using the “survival” and “forestplot” R packages to evaluate the prognostic relevance of G6PD expression with respect to overall survival (OS), disease-specific survival (DSS), progression-free interval (PFI), and disease-free interval (DFI) across different cancer types. Additionally, clinical and transcriptomic data were collected for 319 HCC patients from the TCGA database, 229 HCC patients from the ICGC database, and 177 HCC patients from the GSE14520 database. Both univariate and multivariate Cox regression analyses were performed to identify independent risk factors affecting overall survival in HCC patients.

### Statistical analysis

All statistical analyses were conducted using R version 4.3.1. For continuous data, comparisons between two groups were made using either the independent samples t-test or the Mann-Whitney U test. Univariate and multivariate Cox regression analyses were performed using the “survival” R package to identify independent risk factors. The threshold for defining statistical significance was set at P<0.05 (*P < 0.05, **P < 0.01, ***P < 0.001; ns: not significant).

## Results

### Single-cell atlas and intercellular communication analysis

**Figure 1:**
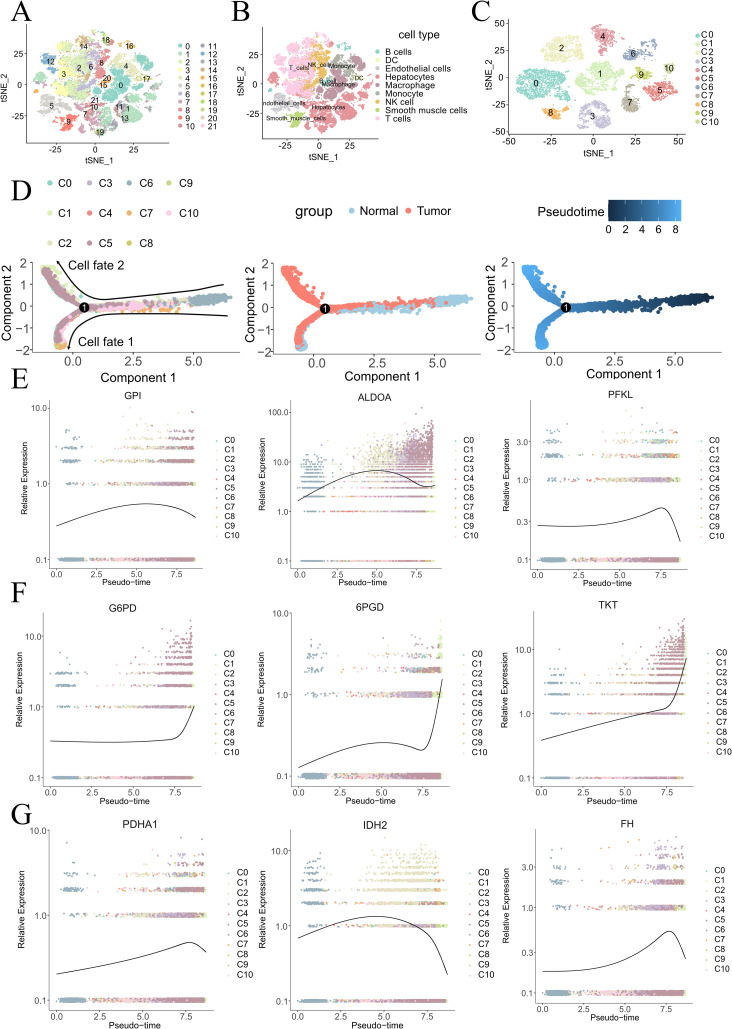
Cell atlas and pseudotime analysis of cancerous and adjacent non-tumor tissues in HCC. t-SNE plots depicting 22 cell populations **(A)** and 9 annotated cell types **(B)** from 7 HCC tissues and paired adjacent non-tumor tissues. **(C)** t-SNE plot of 11 re-clustered hepatocyte populations. Pseudotime analysis **(D)** reveals dynamic expression patterns of glycolysis-related genes **(E)**, PPP-related genes **(F)**, and TCA cycle-related genes **(G)**.

To comprehensively identify the cellular composition and structure of HCC and adjacent non-tumor tissues, we conducted single-cell sequencing analysis on samples from 7 HCC patients. After stringent quality control measures to exclude low-quality cells, a total of 49,324 cells from these tissues for in-depth analysis. Using t-distributed stochastic neighbor embedding (t-SNE) clustering, we organized these cells into 22 distinct clusters (**Figure 1A**). Cell types within the single-cell atlas were annotated using “SingleR” R package, categorizing the cells into 9 types (**Figure 1B**) including smooth muscle cells (ACTA2, TAGLN, RGS5), B cells (CD79A, MS4A1, IGHD), dendritic cells (HLA-DPB1, CLEC9A, CD83), NK cells (NKG7, GNLY, GZMB), T cells (CD3D, CD2, CD3E), monocytes (S100A8, AREG, FCN1), hepatocytes (ALB, TTR, TF), endothelial cells (CLEC4G, ENG, PECAM1), and macrophages (CD68, CD163, CD14) (**Supplementary Figure 1A**). The expression of marker genes in the single-cell map was also displayed (**Supplementary Figure 1B**). **Supplementary Figures 2A, B** shows the cellular composition of both HCC and adjacent non-tumor cells from the 7 cases. We noted a higher proportion of macrophages and a reduced proportion of T cells and NK cells in the tumor tissue compared to the adjacent non-tumor tissue.

### Pseudotime analysis reveals the evolution of glucose metabolism in HCC

To better explore the evolution of glucose metabolism in hepatocytes, we constructed a pseudotime cell trajectory for 11 clusters of hepatocytes (**Figure 1C**) and mapped a bifurcated trajectory representing the development from non-malignant to malignant cells (**Figure 1D**). Cluster 6 (C6), almost exclusively derived from adjacent non-tumor tissue, was identified at the lower right of the trajectory, serving as the initial state’s starting point. This trajectory then bifurcated into two distinct cell fates. Through our pseudotime analysis, we identified three different transformation patterns, colored red (gene expression progressively increasing), blue (gene expression progressively decreasing), and pink (gene expression initially increasing then decreasing) (**Supplementary Figure 2C**).

Enrichment analysis of the biological processes associated with these transformation patterns revealed that the red module was primarily associated with major metabolic processes, including carbohydrate metabolism, fatty acid metabolism, and amino acid metabolism. The blue module was mainly related to immune responses and cell differentiation, while the pink module was associated with protein folding, refolding, and modification.

Mapping glucose metabolism-related genes onto the cell trajectory, we observed trends in the developmental process of the malignancy. Glycolysis-related genes (GPI, ALDOA, PFKL) (**Figure 1E**) and TCA cycle-related genes (PDHA1, IDH2, FH) (**Figure 1F**) both showed trends of initially increasing and then decreasing. In contrast, genes related to thePPP (G6PD, 6PGD, TKT) (**Figure 1G**) exhibited a consistently increasing trend throughout the development of the tumor (28).

### Spatial evolution of glucose metabolism in the HCC Microenvironment

**Figure 2:**
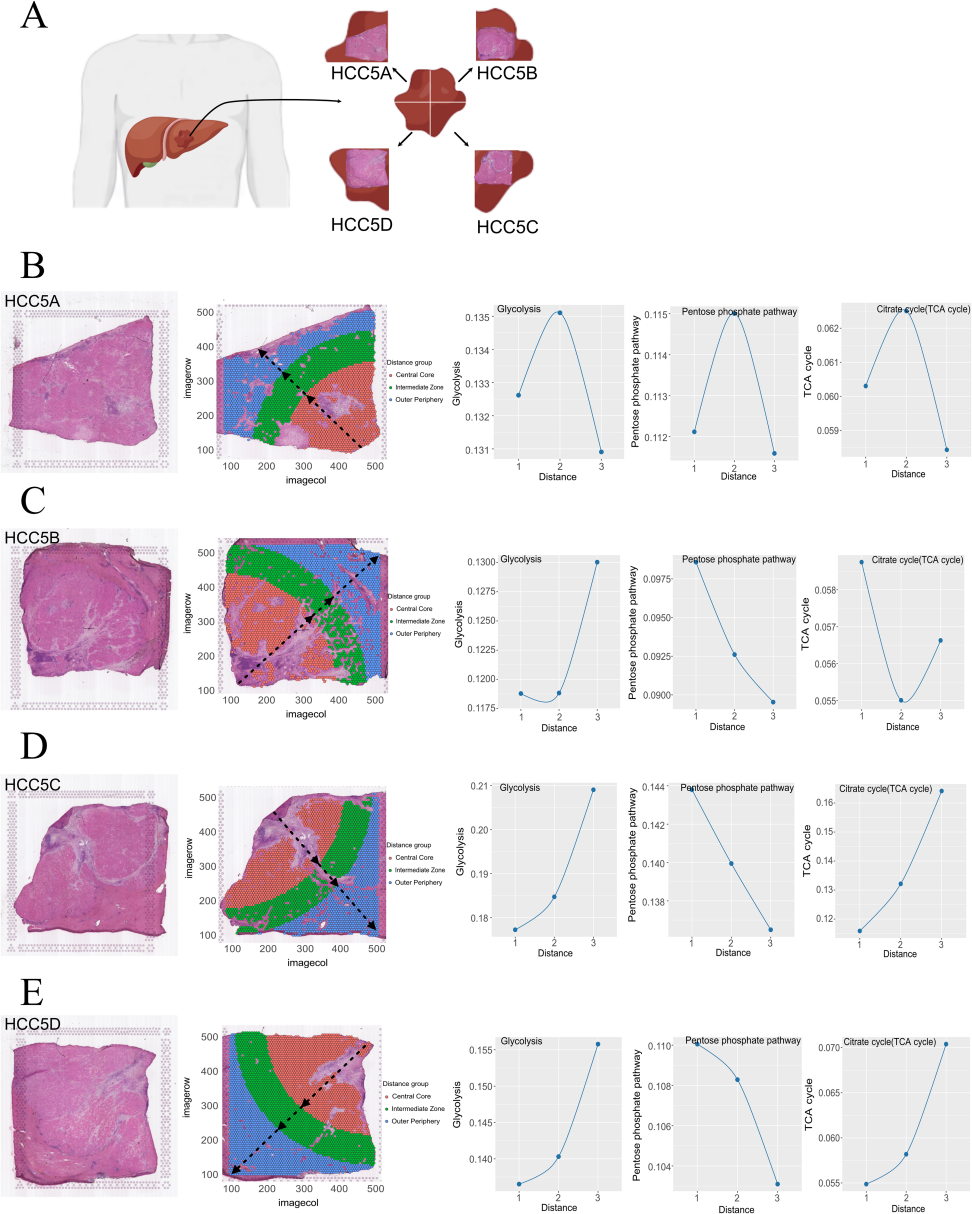
HCC section division diagram **(A)**. Spatial partitioning and glucose metabolism evolution in four cases of HCC **(B–E)**.

Spatial transcriptomics preserves transcriptional data within a spatial context, facilitating the analysis of metabolic pathway activities in localized regions (29). Prior to this study, the spatial dynamics of glucose metabolism within HCC had not been explored. We collected a complete series of tumor sections from HCC patients (HCC5) listed in HRA000437, dividing them into four parts (**Figure 2A**). After removing stromal cells, the sections were further divided into three regions using the tertile method: the central core, intermediate zones, and outer periphery.

Using the scMetabolism tool, we assigned metabolic scores to each cell. For HCC5A (**Figure 2B**), we observed an initial increase followed by a subsequent decrease in the metabolic activity of glycolysis, the PPP, and the TCA cycle from the central core to the outer periphery. In HCC5B (**Figure 2C**), glycolysis and TCA cycle metabolic scores showed a trend of initially decreasing and then increasing towards the out periphery, while the PPP activity consistently decreased. For HCC5C and For HCC5C and HCC5D (**Figures 2D, E**), both glycolysis and TCA cycle activities exhibited an increasing trend, whereas PPP activity consistently decreased from the central core to the outer periphery.

### Knockdown of G6PD inhibits proliferation, migration, and invasion in HCC cells

**Figure 3:**
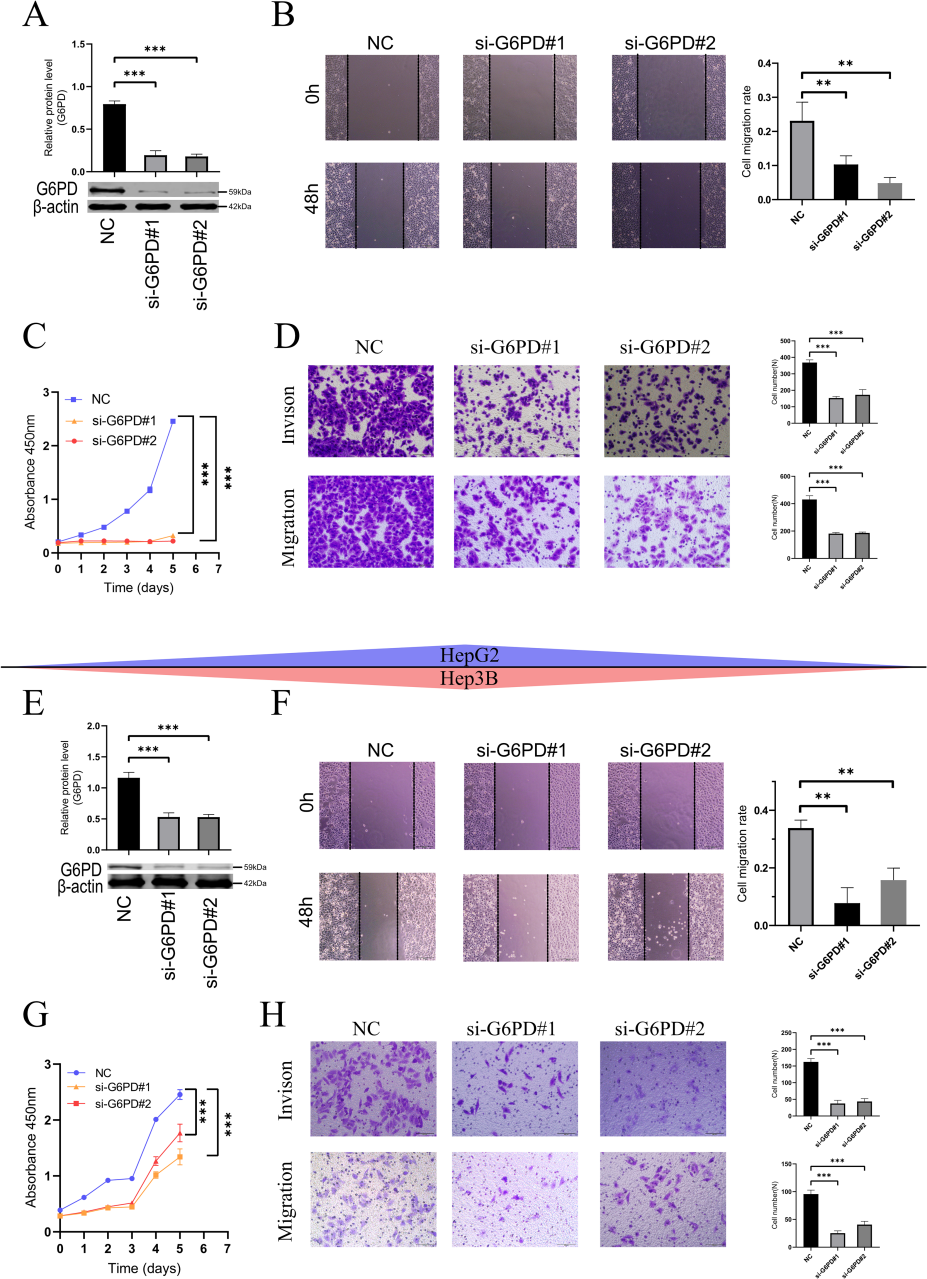
Downregulation of G6PD inhibits proliferation, migration, and invasion in HCC cells (HepG2 and Hep3B). **(A, E)** Western blot (Please refer to **Supplementary Figure 3b** for the detailed WB images) verify the efficiency of G6PD knockdown. **(B, F)** Wound healing assays demonstrate reduced migration rates in HCC cells with lowered G6PD expression. **(C, G)** CCK8 assays indicate that downregulating G6PD inhibits cell proliferation. **(D, H)**. Transwell assays show reduced migration and invasion of HCC cells following G6PD knockdown. (*P < 0.05, **P < 0.01, ***P < 0.001; ns: not significant).

Glucose-6-phosphate dehydrogenase (G6PD) acts as the rate-limiting enzyme in the PPP and plays a crucial role in the development and progression of cancer (30–32). Our study revealed that liver cancer cells (HepG2 and Hep3B) exhibit high endogenous expression of G6PD. Therefore, we constructed G6PD-knockdown HepG2 cells (**Figure 3A**) and Hep3B cells (**Figure 3E**, **Supplementary Figure 3a**).

Wound healing assays (**Figures 3B, F**) revealed that, following G6PD downregulation, both HepG2 and Hep3B cells exhibited slower migration rates. Consistent with these findings, CCK8 assays (**Figures 3C, G**) demonstrated that G6PD knockdown significantly inhibited the proliferation of HepG2 and Hep3B HCC cells. Similarly, in Transwell assays (**Figures 3D, H**), cells with reduced G6PD expression also displayed weaker migration and invasion capabilities.

### Potential mechanisms by which G6PD promotes malignant progression in HCC

**Figure 4:**
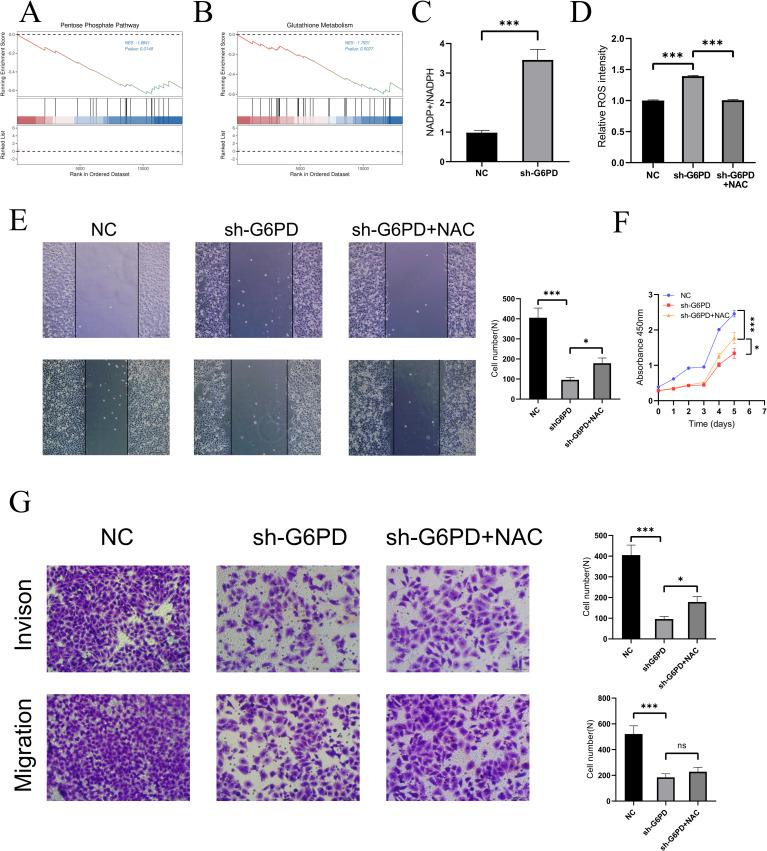
Potential mechanisms by which G6PD promotes proliferation and differentiation in HCC cells. **(A, B)** GSEA enrichment analysis revealed the possible mechanism of G6PD regulation of HCC. **(C)** Downregulation of G6PD leads to increased NADP+/NADPH levels within the HCC cells. **(D)** Downregulation of G6PD leads to increased ROS levels in HCC cells, which could be reversed after NAC treatment. Wound healing assays **(E)**, CCK8 assay **(F)** and transwell assay **(G)** confirmed that ROS can partially regulate the proliferation, migration and invasion of hepatocellular carcinoma. (*P < 0.05, **P < 0.01, ***P < 0.001; ns: not significant).

To preliminarily explore the possible mechanisms by which G6PD promotes hepatocellular carcinoma (HCC) cell proliferation and differentiation, transcriptomic sequencing was performed on the constructed shG6PD HepG2 cells. Notably, GSEA enrichment analysis revealed differential metabolism of the pentose phosphate pathway between the two groups (**Figure 4A**). Additionally, significant differences were observed in glutathione metabolism (**Figure 4B**). We measured NADP+/NADPH levels between the two groups and found that the NADP+/NADPH ratio was higher in the shG6PD group compared to the NC group (**Figure 4C**). As NADPH serves as a reducing equivalent and regulates cellular reactive oxygen species (ROS) stability, we next measured ROS levels and found that downregulation of G6PD led to an increase in ROS in HCC cells (**Figure 4D**). This increase was normalized after treatment with N-acetylcysteine (NAC,1mM). To further investigate whether ROS affects HepG2 cell proliferation, migration, and invasion, we performed wound healing assays (**Figure 4E**), which showed slower migration in the shG6PD cells, and migration was restored following NAC treatment. CCK-8 assays (**Figure 4F**) indicated that shG6PD inhibited HepG2 cell proliferation, which could be partially reversed by NAC treatment. Consistent with the wound healing results, Transwell assays (**Figure 4G**) showed that shG6PD cells had impaired migration and invasion abilities, and these abilities were partially restored after NAC treatment.

### High expression of G6PD correlated with poor prognosis in HCC patients

**Figure 5:**
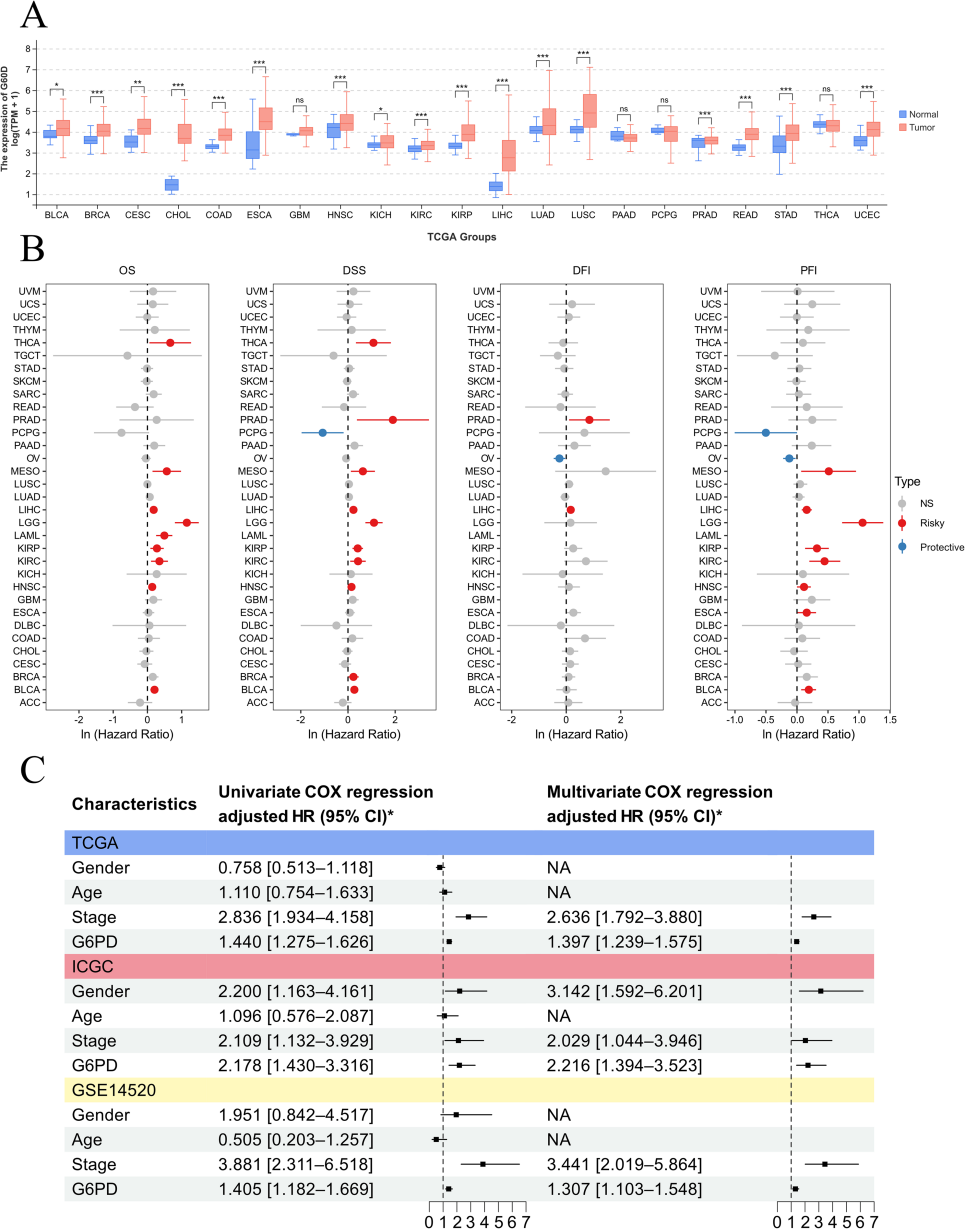
Pan-cancer analysis demonstrating that G6PD is a prognostic biomarker across various cancers. **(A)** Differences in G6PD expression between different tumors and adjacent non-tumor tissues. **(B)** Prognostic analysis of G6PD across various cancers. **(C)** Univariate and multivariate Cox regression analyses reveal that G6PD is an independent risk factor for overall survival in HCC.

To explore the specific expression of G6PD in cancer and its clinical implications, we analyzed the differences in G6PD expression levels between tumor tissues and adjacent normal tissues using data from the TCGA database. Our results (**Figure 5A**) demonstrated that G6PD expression is significantly elevated in multiple cancers, including BLCA, BRCA, CHOL, COAD, ESCA, HNSC, KICH, KIRP, LIHC, LUAD, LUSC, READ, STAD, and UCEC. The abbreviations and full names of tumors can be found in **Supplementary Table 2**.

Subsequently, we utilized univariate Cox regression analysis to clarify the relationship between G6PD levels and patient prognosis in the TCGA cohort (**Figure 5B**). We found that G6PD expression is correlated with prognosis across several cancers, identifying G6PD as a risk factor for overall survival (OS), disease-specific survival (DSS), progression-free interval (PFI), and disease-free interval (DFI) in HCC patients. To further validate the correlation between G6PD expression and prognosis in HCC patients, we collected transcriptomic and clinical data from liver cancer patients across three major databases: TCGA, ICGC, and GEO. Through both univariate and multivariate Cox regression analysis, we established that G6PD is an independent risk factor affecting the overall survival (OS) of HCC patients (**Figure 5C**).

**Figure 6:**
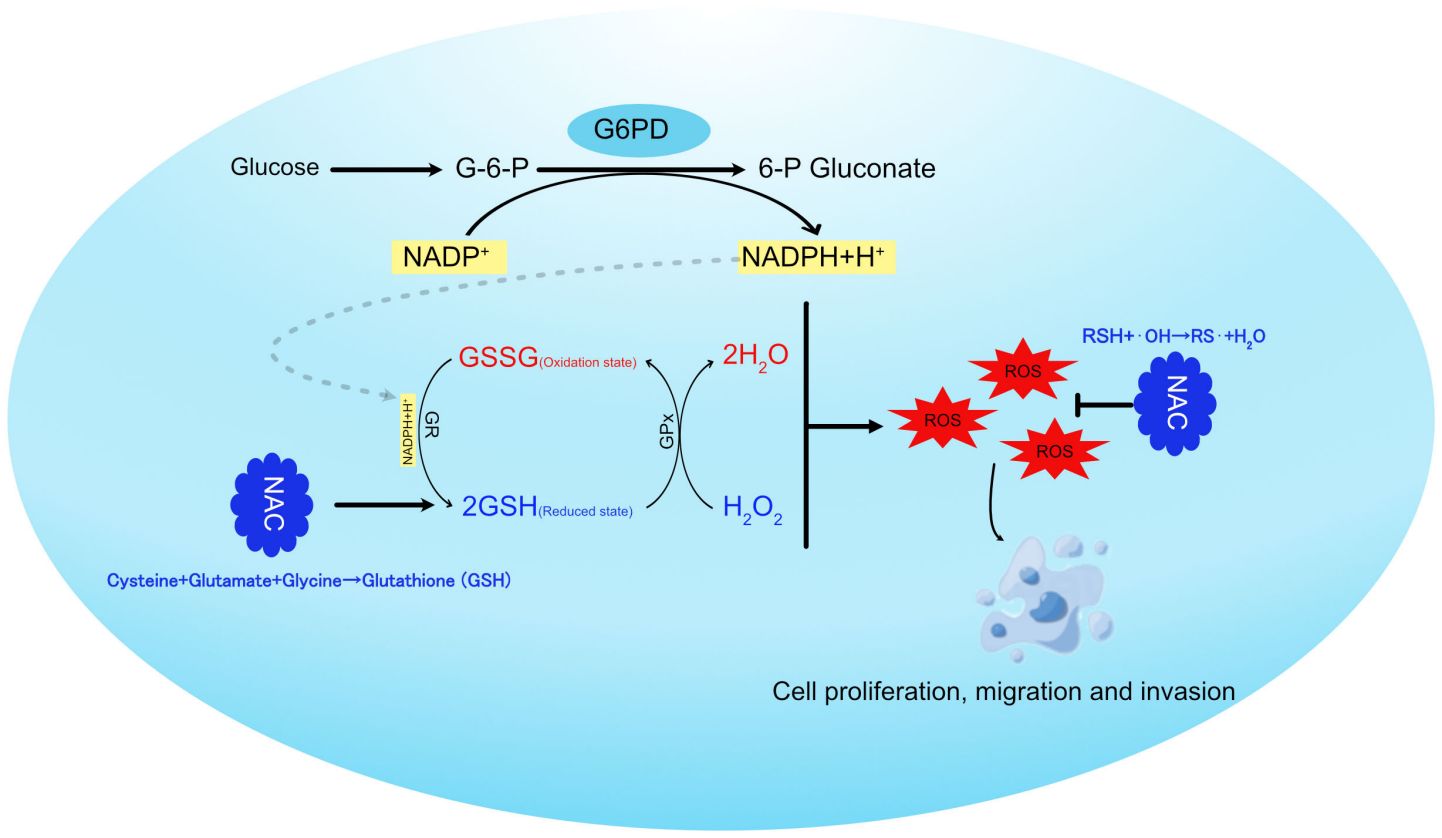
Schematic representation of G6PD regulating glutathione metabolism in hepatocellular carcinoma cells.

In summary, our study suggests that the rate-limiting enzyme of the PPP, G6PD, regulates NADPH production, which in turn modulates glutathione metabolism and ROS generation. This ultimately promotes tumor cell proliferation, migration, and invasion (**Figure 6**).

## Discussion

In this study, through scRNA-seq and ST, we unveiled the dynamic evolution of glucose metabolism in HCC. We described changes in glycolysis, the PPP, and the TCA cycle from both temporal and spatial perspectives. Temporally, we observed an initial increase followed by a decrease in glycolysis and TCA cycle-related genes, whereas PPP-related genes consistently increased. Spatially, from the core to the periphery, the metabolic activity of glycolysis shows a gradual increase, while the activity of the PPP gradually decreases. The metabolic changes observed in HCC5A are inconsistent with those in the other samples; we suspect this is because HCC5A is composed almost entirely of HCC cells from the core region, with little to no representation of HCC cells from the tumor periphery. Additionally, we confirmed through cellular experiments that G6PD, a key enzyme in the PPP, can regulate the proliferation, migration, and invasion of HCC cells and preliminarily analyzed potential mechanisms by which G6PD facilitates malignant progression in HCC.

Metabolic reprogramming in cancer cells allows for the adjustment of intracellular metabolic pathways and the distribution of metabolic products, thereby modulating cell function and physiological states (33, 34). The growth and proliferation of cancer cells demand substantial energy and resources (35). Reprogramming glucose metabolism helps adjust glucose turnover within cells, enabling more efficient energy production and synthesis of necessary macromolecules, thereby supporting malignant proliferation and differentiation (36–39). Our findings suggest that enhanced glycolysis in tumor cells presents an initial rise followed by a decline, possibly due to several factors: 1. Fast ATP production by glycolysis, which compensates for the inhibited mitochondrial oxidative phosphorylation due to local hypoxia or other factors (40). 2. Intermediate metabolic product accumulation from glycolysis, such as pyruvate, which can be used for lipid synthesis (41). 3. Acidification of the microenvironment by lactate produced through glycolysis, facilitating tumor invasion and immune evasion (42, 43). In the late stages of tumor development, the decline in glycolysis-related gene expression could be linked to extreme environmental stresses (like severe hypoxia or ischemia) or a reduced metabolic state akin to dormancy due to internal or external pressures. We observed a similar phenomenon at the spatial level, where the glycolytic metabolic activity in the tumor core region was less vigorous than in the tumor periphery. This may also be related to the extreme stress environment and the internal and external pressures that lead to tumor cell dormancy.

In clinical research, inhibition of G6PD has emerged as a promising strategy for cancer therapy (44). G6PD is a pivotal enzyme in the glycolytic pathway, primarily producing NADPH via the pentose phosphate pathway (PPP), which is crucial for maintaining cellular redox balance and influencing cellular processes such as growth, differentiation, and apoptosis (45). The upregulation of G6PD is not only associated with the enhanced proliferation, migration, and invasion of tumor cells but also promotes epithelial-mesenchymal transition (EMT) and metastasis through the modulation of cellular redox status (46). Furthermore, G6PD is closely linked to chemotherapy resistance in various cancers, as it aids tumor cells in counteracting oxidative stress and DNA damage induced by chemotherapeutic agents (47). Several small-molecule G6PD inhibitors, such as 6-aminohexose (6-AN) and dehydroepiandrosterone (DHEA), have demonstrated potential to suppress tumor growth (48–50). In experimental settings, DHEA has been shown to enhance the sensitivity of certain tumor cells to conventional chemotherapeutic agents like paclitaxel and doxorubicin, and even to counteract chemotherapy resistance in tumors (50, 51). However, inhibition of G6PD not only impacts tumor cell metabolism but may also have detrimental effects on normal cells. G6PD inhibition results in decreased NADPH levels, thereby weakening the cellular antioxidant capacity and increasing oxidative stress and DNA damage. This effect could lead to damage in normal cells, with particularly pronounced effects on organs such as the liver and bone marrow (52). Currently, there is no consensus regarding the optimal timing and administration methods for G6PD inhibitors in clinical settings. Given the multifaceted role of G6PD in tumor cells, we propose that simple G6PD inhibition may be insufficient to fully suppress tumor growth. However, combining G6PD inhibitors with chemotherapeutic agents could offer a novel treatment strategy for cancer patients. Presently, the administration of G6PD inhibitors has certain drawbacks. For instance, 6-AN and DHEA are typically administered orally; however, their significant side effects and limited targeting capabilities significantly hinder their broad clinical application (53). With the advancement of nanomaterials, strategies involving local delivery or encapsulation of G6PD inhibitors in nanoparticles may allow for targeted delivery to tumor cells, minimizing systemic side effects while enhancing therapeutic efficacy (54). In conclusion, G6PD, as a critical metabolic regulator, is emerging as a novel target for cancer therapy. While clinical research is still in its early stages, the therapeutic opportunities and challenges it presents warrant further exploration.

In our study, the metabolic activity of the PPP consistently increased over time and was more pronounced in the core and intermediate areas of tumors. The PPP, alongside glycolysis, generates ribose-5-phosphate and NADPH, which can reduce excessive ROS in cells, maintaining internal cellular balance and normal growth conditions (55). However, when this balance is disrupted, ROS can promote tumor development by increasing genetic instability, but post-tumor establishment, it can limit cancer cell survival and growth (56, 57). G6PD is the rate-limiting enzyme in the PPP. Studies have shown that PBX3 binds to the G6PD promoter, stimulating the PPP in colorectal cancer and increasing the production of nucleotides and NADPH, thereby promoting the biosynthesis of nucleic acids and lipids while reducing oxidative stress (58). Consistent with our findings, knocking down G6PD significantly inhibited proliferation, migration, and invasion of HepG2 and Hep3B cells, likely linked to the production of ribose-5-phosphate and NADPH (59). NADPH provides the reducing equivalents necessary to convert oxidized glutathione (GSSG) back to its reduced form (GSH), thereby maintaining cellular antioxidant capacity. In turn, GSH plays a crucial role in mitigating the excessive accumulation of ROS (60). Additionally, Min Li et al., through transcriptomic analysis, demonstrated that Aldob directly binds to G6PD and inhibits its activity, thereby suppressing the PPP and exerting a novel tumor-suppressive role in HCC (61). Therefore, G6PD inhibition represents a viable strategy for cancer treatment.

In conclusion, our integrated scRNA-seq and ST analysis revealed the metabolic evolution of glycolysis, PPP, and TCA cycle in HCC cells, confirming G6PD’s regulatory role on tumor aggressiveness and its potential as a prognostic marker and therapeutic target in HCC. Nonetheless, our study has inherent limitations: single-cell and spatial transcriptomic data were not from the same patient samples; and while bioinformatics analysis and experimental validations were employed, more extensive experimental validations are needed to corroborate these findings. Although our study utilized two liver cancer cell lines for validation, it still cannot replicate the heterogeneity of liver cancer. Further experimental validation using primary cell cultures is needed. Finally, our study also lacks further *in vivo* exploration, particularly animal studies. Future work will focus on recruiting a larger cohort of HCC patients and conducting animal studies to overcome these limitations and employ diverse methods to rigorously analyze glucose metabolism alterations in HCC.

## Data Availability

The original contributions presented in the study are included in the article/**Supplementary Material**. The TCGA dataset was downloaded from the GDC portal (https://portal.gdc.cancer.gov/); The GSE14520 and GSE149614 datasets were downloaded from the GEO database (https://www.ncbi.nlm.nih.gov/geo/query/acc.cgi?acc=GSE14520 and https://www.ncbi.nlm.nih.gov/geo/query/acc.cgi?acc=GSE149614); HRA000437 reported in this article can be acquired from the Genome Sequence Archive (GSA-Human: HRA000437) and is publicly accessible at https://ngdc.cncb.ac.cn/gsa-human/browse/HRA000437. Further inquiries can be directed to the corresponding author(s).
