# Supplementary material for: Single-cell sequencing and spatial transcriptomics reveal the evolution of glucose metabolism in hepatocellular carcinoma and identify G6PD as a potential therapeutic target
Source: Front Oncol. 2025 Mar 25;15:1553722. doi: 10.3389/fonc.2025.1553722 (PMC11975570; doi:10.3389/fonc.2025.1553722)
Supplement: Supplementary file 1 [file DataSheet1.pdf]

Supplementary information

Supplement Table 1 Primer sequence

Name	5'-3'(S/F)	5'-3'(AS/R)
si-G6PD#1	GCCGTGTACACCAAGATGA	UCAUCUUGGUGUACACGGCTT
si-G6PD#2	GUGCUGAGAUUUGCCAACATT	UGUUGGCAAAUCUCAGCAACATT
β-Actin	AGCACAGAGCCTCGCCTTT	ATCATCATCCATGGTGAGCTGG
G6PD	ACATGAATGCCCTCCACCTG	ATGCGGTTCCAGCCTATCTG

Supplement Fig. 1

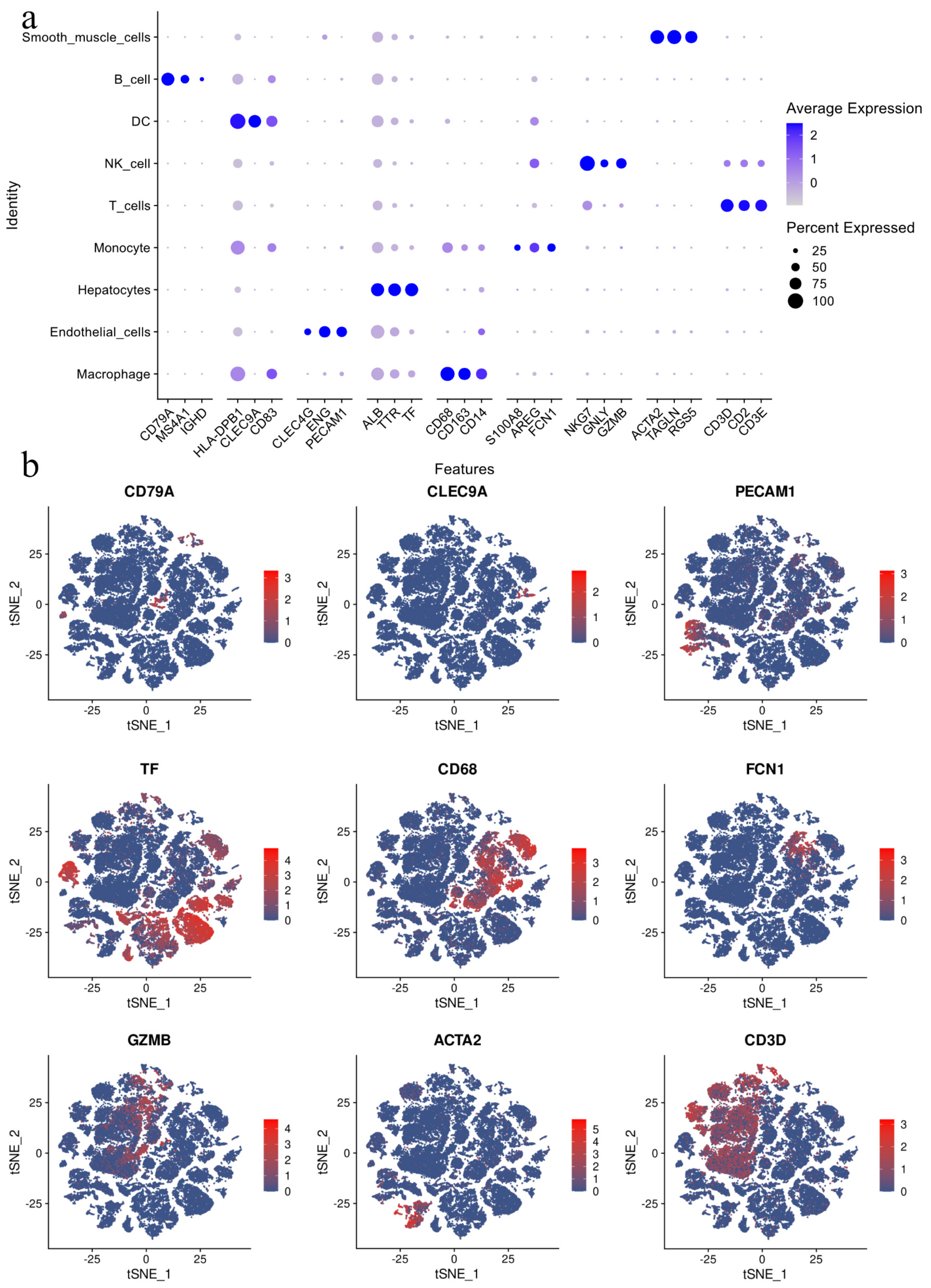

Supplement Fig. 1: (a) Bubble chart showing marker gene expression in major cell types. (b) Distribution of characteristic genes from each cell cluster across all cells.

Supplement Fig. 2

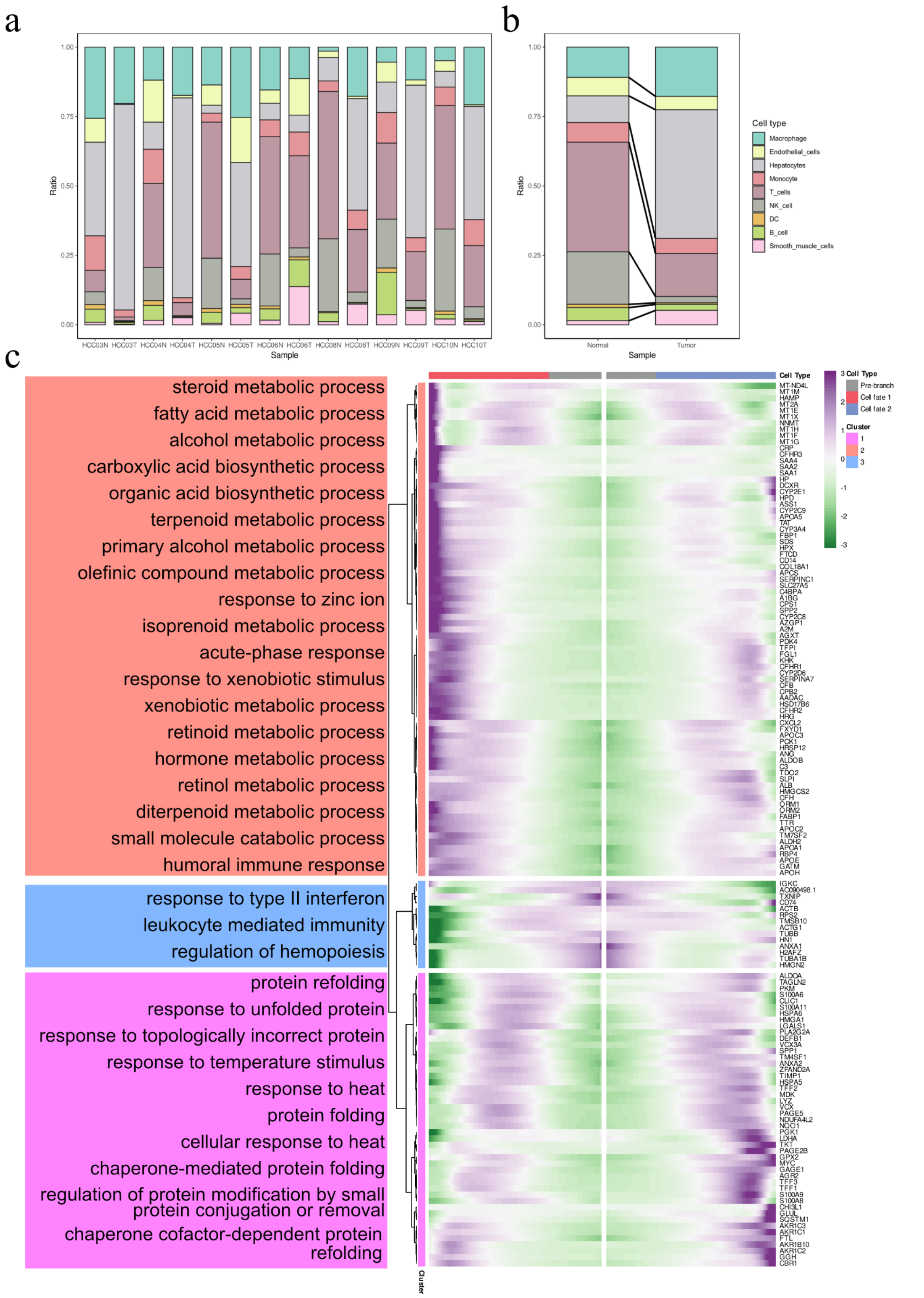

Supplement Fig. 2: Composition of cells in different samples and groups. (a) Cellular composition across various samples. (b) Cellular composition across different groups. (c) Pseudotime analysis revealing three transformation patterns and enrichment analysis of these transformation patterns.

Supplement Fig. 3

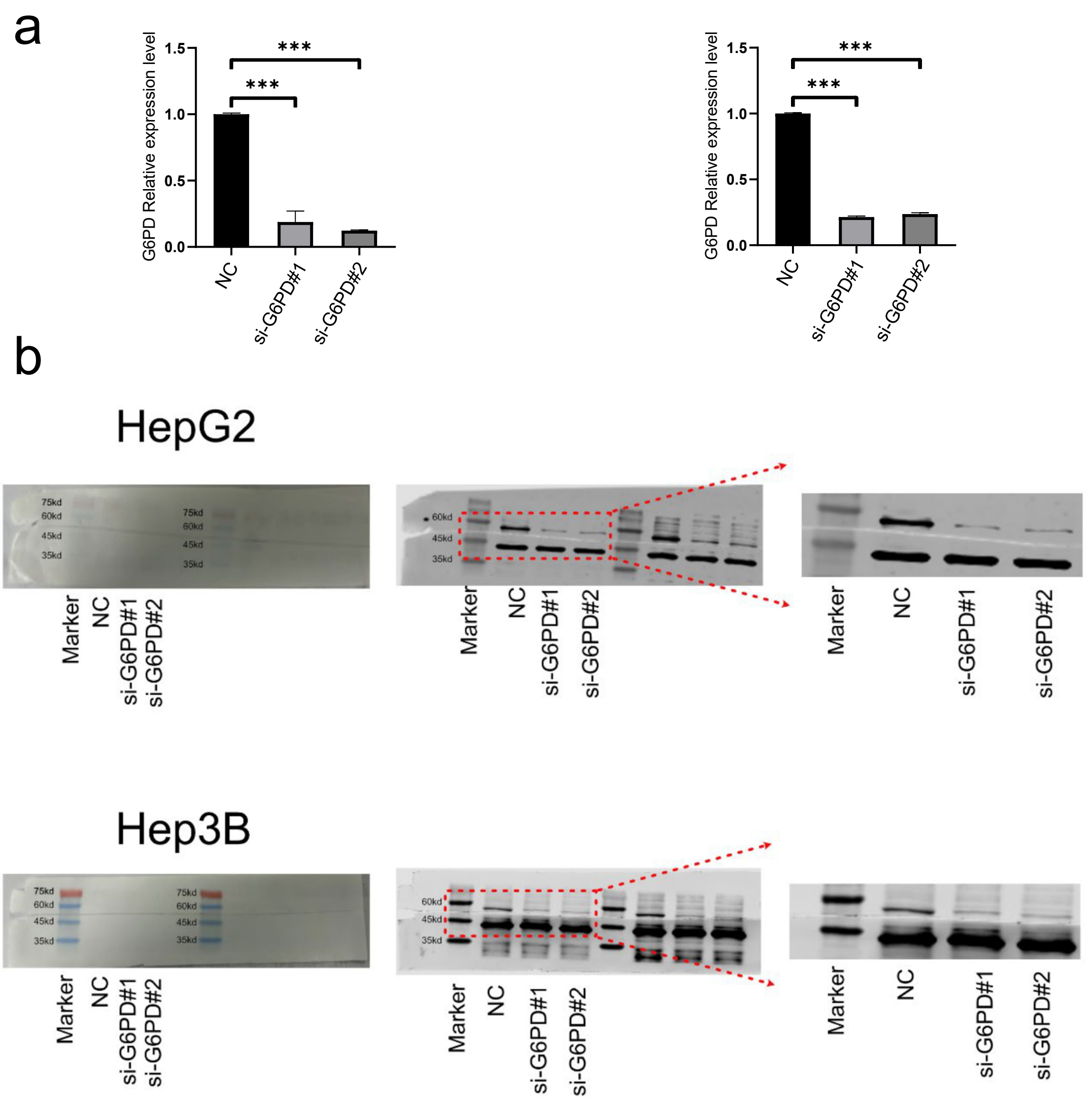

Supplement Fig. 3: (a) RT-qPCR verify the efficiency of G6PD knockdown. (b)WB whole membrane images of G6PD knockdown in HCC cells

Supplement Table 2 Abbreviations and full names of different tumor types

Types of cancer	abbreviate
Adrenocortical Carcinoma	ACC
Bladder Urothelial Carcinoma	BLCA
Breast Invasive Carcinoma	BRCA
Cervical Squamous Cell Carcinoma and Endocervical Adenocarcinoma	CESC
Cholangiocarcinoma	CHOL
Colorectal Adenocarcinoma	COAD
Diffuse Large B-cell Lymphoma	DLBC
Esophageal Carcinoma	ESCA
Glioblastoma Multiforme	GBM
Head and Neck Squamous Cell Carcinoma	HNSC
Kidney Chromophobe	KICH
Kidney Renal Clear Cell Carcinoma	KIRC
Kidney Renal Papillary Cell Carcinoma	KIRP
Acute Myeloid Leukemia	LAML
Lower Grade Glioma	LGG
Liver Hepatocellular Carcinoma	LIHC
Lung Adenocarcinoma	LUAD
Lung Squamous Cell Carcinoma	LUSC
Mesothelioma	MESO
Ovarian Cancer	OV
Pancreatic Adenocarcinoma	PAAD
Pheochromocytoma & Paraganglioma	PCPG
Prostate Adenocarcinoma	PRAD
Rectal Adenocarcinoma	READ
Sarcoma	SARC
Skin Cutaneous Melanoma	SKCM
Stomach Adenocarcinoma	STAD
Testicular Germ Cell Tumor	TGCT
Thyroid Cancer	THCA
Thymoma	THYM
Uterine Corpus Endometrial Carcinoma	UCEC
Uterine Carcinosarcoma	UCS
Uveal Melanoma	UVM
